# Effects of a 3-week foam rolling warm-up intervention on ankle dorsiflexion measurements and jumping performance in young rhythmic gymnasts

**DOI:** 10.1186/s13102-026-01575-2

**Published:** 2026-02-10

**Authors:** Giacomo Belmonte, Antonino Patti, Irene Rosa Di Mitri, Marco Gervasi, Eugenio Formiglio, Eneko Fernández-Peña, Ewan Thomas, Antonino Bianco

**Affiliations:** 1 Sport and Exercise Sciences Research Unit, Department of Psychology, Educational Science and Human Movement, University of Palermo, Palermo, 90144 Italy; 2 Department of Biomolecular Sciences, Division of Exercise and Health Sciences, University of Urbino Carlo Bo, Urbino, 61029 Italy; 3 Department of Physical Education and Sport, University of the Basque Country UPV/EHU, Vitoria-Gasteiz, 01007 Spain

**Keywords:** Myofascial release, Rhythmic gymnastics, Jumping performance, Biomechanics

## Abstract

**Background:**

Few studies have analyzed the medium- to long-term effects of warm-up protocols on biomechanical and performance parameters in athletes. This study evaluated the effects of a 3-week foam rolling (FR) protocol integrated into the initial phase of the warm-up on ankle dorsiflexion parameters and jumping performance through the countermovement jump test (CMJ) in young rhythmic gymnasts.

**Methods:**

Twenty-six female young rhythmic gymnasts who met the inclusion criteria participated in the study and were divided into the FR group (*n =* 12; Age = 13.2 ± 2.59 years; Height = 152.3 ± 13.34 cm; Weight = 39.9 ± 11.22 kg) or the C (Control) group (*n =* 14; Age = 13.4 ± 2.17 years; Height = 149.5 ± 13.33 cm; Weight = 40.6 ± 10.14 kg). An initial evaluation (T0) and an evaluation after the 3-week FR warm-up protocol were conducted. An inertial sensor was used to analyze ankle dorsiflexion using the following parameters: range of motion (ROM) (°), angular velocity (°/s), and fluency index (0–1). An optical detection sensor detected the jump height and flight time of the CMJ test.

**Results:**

Significant differences were found between T0 and T1 in the FR group and between the FR group and the C group at T1. Significant between-subject interaction effects (F = 32.58; *p <* 0.001; ηp^2^ = .576) and group-by-time interaction effects (F = 9.73; *p <* 0.01; ηp^2^ = .288) were found. Specifically, significant enhancements in CMJ performance (*p <* 0.001) and angular dorsiflexion velocity of the right ankle (*p <* 0.05) were noted after the intervention in the FR group.

**Conclusions:**

Our results suggest that a 3-week FR intervention incorporated into the warm-up routine of young rhythmic gymnasts led to an increase in jump performance, but not in ankle dorsiflexion ROM parameters. Further studies are necessary to investigate the effects of warm-up on biomechanical parameters using longer FR protocols.

**Trial registration:**

NCT07113249. Registration date: 02/08/2025.

## Introduction

Rhythmic gymnastics (RG) is an Olympic sport discipline characterized by individual or group competitions [1, 2]. These competitions are conducted using various equipment, including ropes, hoops, balls, clubs, and ribbons [2]. The particular exercises showcase the high level of athletic and artistic training of the gymnasts through the performance of technical exercises such as jumps and rotations around the longitudinal axis (pivots), accompanied by music [3]. This type of sport is known for early specialization, which subjects athletes to intense training loads from as 4 or 5 years old [4]. Indeed, high-level rhythmic gymnasts undergo training sessions lasting up to 18–20 h per week from the age of 7, focusing on muscle flexibility, balance and coordination, muscle strength, and cardiovascular fitness [5]. Such a high training load in such young athletes can increase the incidence of injuries, especially if the workload is not well balanced, predisposing them to overtraining or even abandoning the sport activity [6, 7]. In this regard, a study conducted by Gulati et al. in 2022 analyzed the most frequent injuries in RG, finding that overuse injuries were the most common (76.7%), especially foot (24.9%) and ankle (15.5%) injuries [8]. In line with these results, another study conducted by Edouard et al. analyzed the most frequent injuries in gymnastics in the years leading up to the 2008, 2012, and 2016 Olympics [9]. In this study, the ankle (22%) was found to be the site most affected by injuries, and ankle sprains (35%) were the most frequent injury in gymnastics [9]. Ankle sprains pose a significant risk to individuals who engage in a wide range of activities and sports [10]. Ankle joints can undergo excessive inward stress during certain sports activities, leading to chronic ankle instability (CAI) and recurrent ankle sprains [10, 11]. For this reason, the scientific literature explored effective strategies for injury prevention and optimizing athletic performance, such as various warm-up protocols before training and competition [12, 13]. Warm-up protocols represent a fundamental component of athletic preparation and may include low-intensity aerobic activity, static and dynamic stretching, or bodyweight movements [14, 15]. To further optimize performance, these sessions often incorporate sport-specific exercises, either in isolation or in combination [16, 17]. Performance optimization is strongly influenced by a range of thermal and non-thermal factors. In addition to vascular and metabolic changes, important neuromuscular adaptations, such as acceleration of nerve conduction velocity and enhancement of motor unit recruitment, modulate the force–velocity relationship, directly refining the neuromuscular control necessary for high-intensity and high-precision athletic activities [16–18]. Recently, the term post-activation performance enhancement has been used to define the acute increase in athletic performance measures following warm-up, achieved through high-intensity, specific, and well-structured exercise protocols [18–20]. In particular, the warm-up phase was also examined in the RG, as reported in the study by Guidetti et al. [21]. This study analyzed the warm-up routines performed by elite and subelite RG athletes [21]. Slow running was identified as the most commonly used initial part by both groups, followed by various flexibility exercises and specific jumps [21]. In recent years, foam rolling (FR) has become a widely used tool in sports contexts, both for preparation for training and competitions and to accelerate post-exercise recovery [22, 23]. Indeed, FR is recognized for increasing joint ROM without compromising muscle strength and, therefore, athletic performance [24, 25]. In this regard, Konrad et al. in 2022 analyzed the acute effects of FR within different warm-up protocols: “intense warm-up + FR,” “FR + intense warm-up,” or “FR only” [26]. The authors described a 2-min FR protocol for unilateral application to the hamstrings, starting with the left leg, asking each participant to return to the starting position every 4 s [26]. However, FR intervention produced the same increased effect on joint ROM without compromising jumping performance, regardless of the type of warm-up studied [26]. Similarly, Lopez-Samanes et al. analyzed the acute effects of two warm-up protocols: dynamic warm-up or self-myofascial release with FR on sports performance parameters and joint ROM in tennis players [27]. The athletes performed 8 min of FR unilaterally on the quadriceps, hamstrings, glutes, and gastrocnemius for 60 s on both legs, for a total of 8 min [27]. The FR protocol had significant acute effects on joint ROM and a moderate impact on jump performance (CMJ) [27]. However, the long-term impact of FR interventions in the warm-up phase has been little explored in sport activities and the results are conflicting. So, this study aimed to evaluate the effectiveness of a 3-week Foam Rolling protocol in warm-up on ankle dorsiflexion measurements and jumping ability in elite young rhythmic gymnasts.

## Methods

### Experimental set-up

**Fig. 1:**
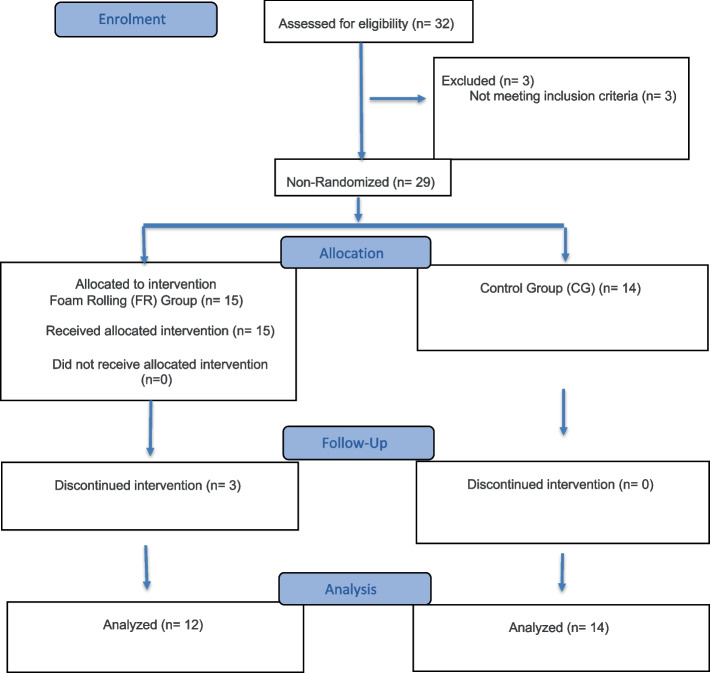
The TREND diagram shows the flow of participants through each stage of the non-randomized trial

**Fig. 2:**
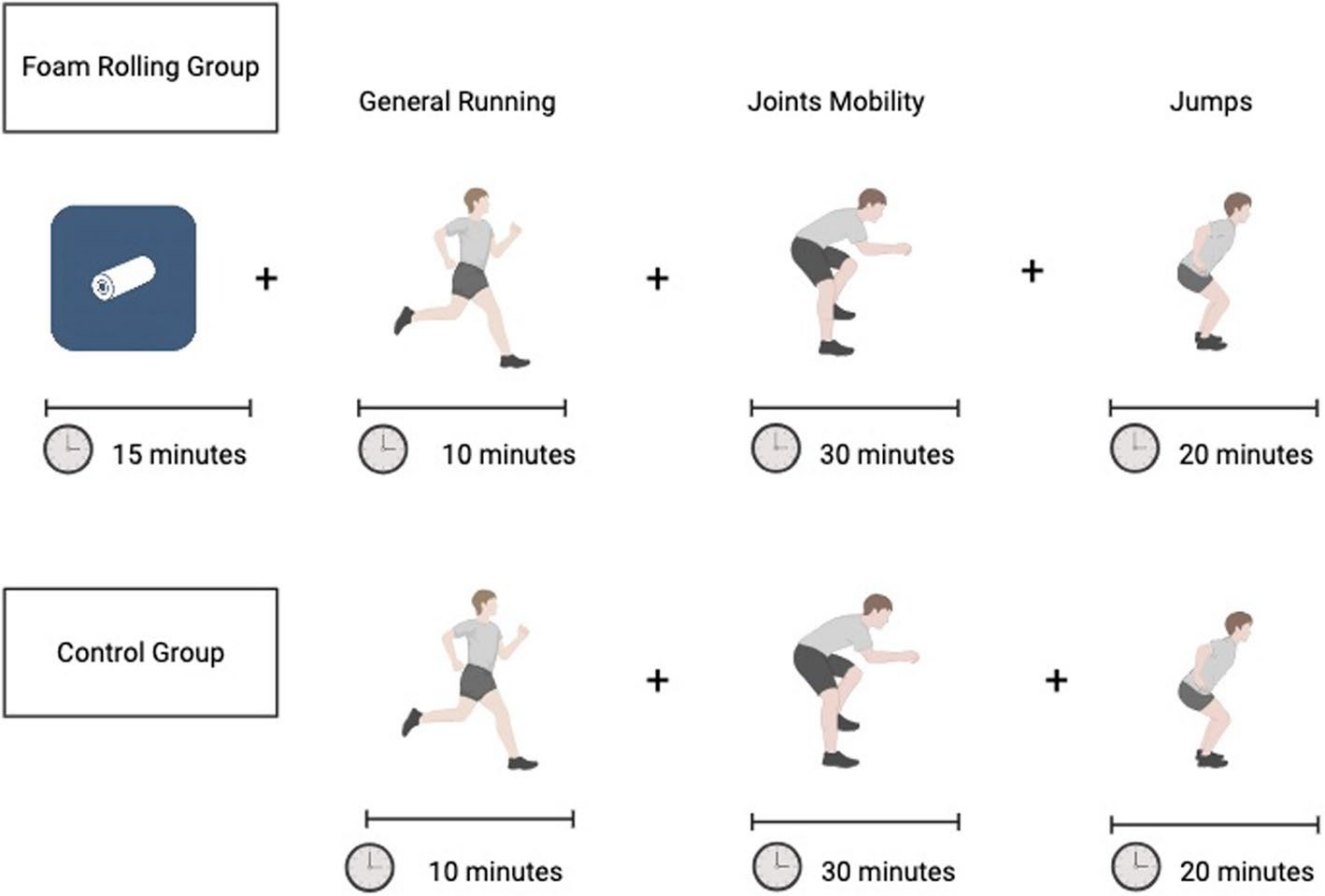
The Experimental Warm-Up protocols conducted in both groups

A non-randomized controlled design was conducted to evaluate the effects of FR in a warm-up protocol on the calf muscles and foot-rolling on the plantar surface. This study design was adopted to maintain the integrity of the training groups within the elite youth environment and to avoid disrupting the established training protocols. The TREND flow diagram (Fig. 1) illustrates the progression of participants through each phase of the non-randomized trial [28]. The measurement sessions were conducted before the interventions (T0) and after 3 weeks (T1) by the same rater, who was uninformed of the aim of the study. Initially, an investigation was conducted on the warm-up usually performed, and it was unified for every participant. Subsequently, two types of warm-up protocols were developed and administered, differing only in the FR section, as shown in Fig. 2. Hence, the participants were divided into two groups: a) an FR protocol placed at the beginning of the warm-up for three days for week on the muscles responsible for dorsal flexion of the foot (gastrocnemius, soleus, and foot muscles); and b) the Control group that underwent the same warm-up protocol without the FR phase. All interventions and measurements were carried out at the same time of the day, and without wearing shoes. All participants were tracked regarding training volume and were evaluated during the same competitive period. According to the study by Patti et al., each FR session lasted 60 s, with three sets completed for a total of 180s [29]. This protocol was chosen both for the FR areas treated and for the measurements evaluated in the study [29]. Before performing the warm-up procedure, two sessions of familiarization were administered to the respective groups with the protocols.

### Participants

At the start of the study, thirty-two rhythmic gymnasts were evaluated for initial screening. However, three subjects were excluded because they did not meet the study inclusion criteria. The following inclusion criteria were used: age between 9 and 17 years, active participation in competitive training programs (at least 10 h per week), at least one year of competition experience, and no ankle injuries in the six months prior to the study. Those who presented injuries at the time of the study or who had not yet recovered from previous injuries, and those who were unable to maintain a regular training routine during the study period, were excluded. Subsequently, three subjects who did not complete the intervention were further excluded. These subjects were excluded due to dropouts and absences. As a result, the sample consisted of twenty-six participants. All participants were young female rhythmic gymnasts. The average age of participants was 13.3 years (SD: 2.32); the average weight was 40.3 kg (SD: 10.44); and the average height was 150.8 cm (SD: 13.15). The TREND flowchart (Fig. 1) was used to ensure that the evaluation of study participants was conducted clearly.

### Measurements

#### Ankle dorsiflexion parameters

An inertial sensor with Bluetooth data transmission was used, along with its specific software, to collect data related to ankle joint dorsiflexion movements accurately and reproducibly (Beyond Inertial, Motusech, Roma, Itay) [30]. This inertial sensor possesses an internal sampling rate of up to 1000 Hz; a 3-axis gyroscope with programmable FSR of ± 250dps, ± 500dps, ± 1000dps, and ± 2000dps; a 3-axis accelerometer with programmable FSR of ± 2 g, ± 4 g, ± 8 g, and ± 16 g; and a 3-axis compass with a wide range to ± 4900μT. Each participant was fitted with an elastic band supplied with the sensor in the center of the foot. The starting position was standardized with the subjects sitting on a medical bed with the popliteal cord attached to the edge of the bed, forming a 90° angle. Subsequently, each participant was asked to actively perform a maximum dorsiflexion movement of the ankle. Participants were asked to perform a maximum active dorsiflexion task to evaluate functional ankle mobility. Active dorsiflexion movement was prioritized to reflect the voluntary end-range control necessary for technical execution in rhythmic gymnastics. A laser level was used to ensure the correct starting position of the foot during the dorsiflexion movement of both ankles. The following parameters were considered for the analysis: a) dorsiflexion ROM (°); b) angular velocity of ROM (°/s); c) fluency index (0–1) [29, 30]. Three measurements were taken for each parameter, and an average was calculated for data analysis.

#### Counter-Movement Jump (CMJ) test

An optical system with two bars was used to perform the CMJ parameter (Optojump Next; Microgate SRL; Bolzano, Italy). This system allows flight and contact times to be measured during a series of jumps with great accuracy [31]. It is an optical detection system that consists of a transmitter bar and a receiver bar, each equipped with 96 LEDs. The system detects any interruptions and measures their duration. This enables the measurement of flight and contact times during a series of jumps. Optojump Next, along with its specific software, was used on each participant barefoot, and the best jump out of three, with two two-minute intervals, was selected for statistical analysis. During the test, participants performed a counter movement jump (CMJ) starting from a standing position with their hands on their hips. They quickly bent their knees to form an angle of approximately 90° and then jumped as high as possible in the subsequent phase of maximum extension [32]. Participants were asked to lift themselves off the ground with their knees and ankles fully extended to avoid overestimating their jump values. Jumps that did not comply with the correct CMJ protocol were excluded. The values used for statistical analysis were flight time (s) and jump height (cm).

#### Intervention: Foam Rolling (FR)

Participants were instructed in the FR protocol by an expert in sports science, who supervised the intervention throughout all sessions. Two different types of expanded polypropylene foam rollers were used: one measuring 33 × 14 × 14 cm for the posterior leg muscles, particularly the gastrocnemius and soleus muscles, and a smaller one measuring 15 × 5.3 × 5.3 cm for the sole. Before starting the treatment period, two familiarization sessions were administered by the same operator. Regarding the protocol used, in line with Patti et al. [29] and Kasahara et al. [25], three sets of 60 s, with a 30-s break between sets, before the warm-up and activation session, with a foam rolling protocol, were administered for 3 weeks at a frequency of twice a week. During each FR session, a continuous movement was initially executed from the gastrocnemius muscles to the soleus with the first foam roller. The second foam roller was then used on the muscles of the sole to treat the flexor muscles of the big toe and toes. A metronome was used to perform one complete repetition every 2 s to standardize the rolling speed for each participant. The FR intervention protocol was included at the beginning of the training sessions, in addition to the warm-up protocols most used in rhythmic gymnastics [21], as shown in Fig. 2.

### Statistical analysis

All data were initially extracted from the relevant software and transcribed into an Excel spreadsheet (Microsoft Corporation, Redmond, WA, USA, version 16.32). Subsequently, statistical analysis was carried out using Jamovi software (version 2.3.28). To assess the normality, the Shapiro–Wilk test was performed, and all data were transcribed as mean and standard deviation (SD). An independent Student's T-test was used to evaluate differences in anthropometric characteristics. A repeated measures ANOVA was performed to examine the effects of FR in addition to a warm-up protocol on ankle dorsiflexion parameters and CMJ ability. The analysis evaluated the evolution over time (T0-T1) of two different interventions (FR-C group), assessing both their effects and interactions. For the effect size, ηp^2^ was used and classified as: less than 0.01 as a small effect; between 0.02 and 0.1 as a moderate effect; and greater than 0.1 as a large effect [33]. Tukey's post-hoc analysis for multiple comparisons was performed to analyze the significant difference between tests over time and between groups. The significance level was set at *p <* 0.05.

## Results

**Fig. 3:**
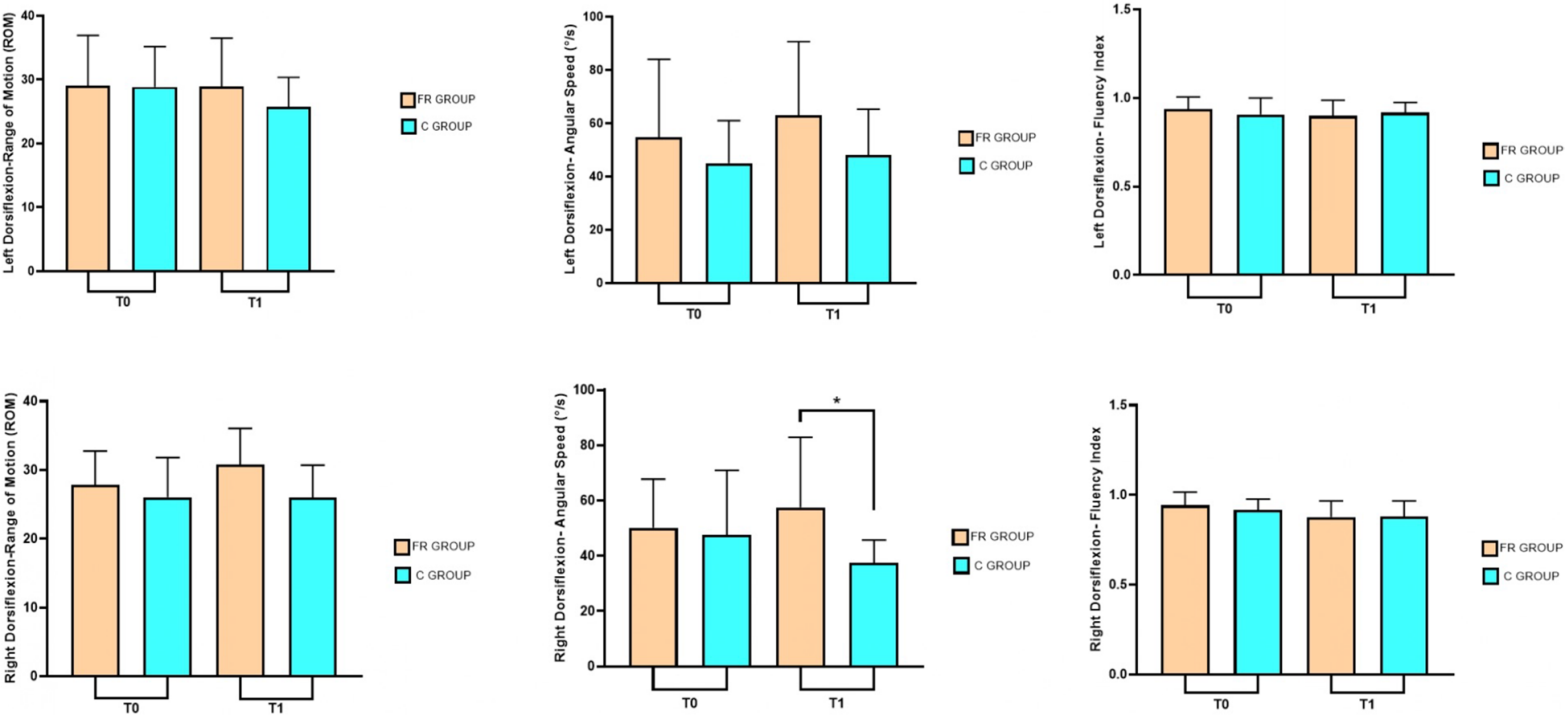
Differences in ankle dorsiflexion parameters before (T0) and after (T1) the interventions for both groups

**Fig. 4:**
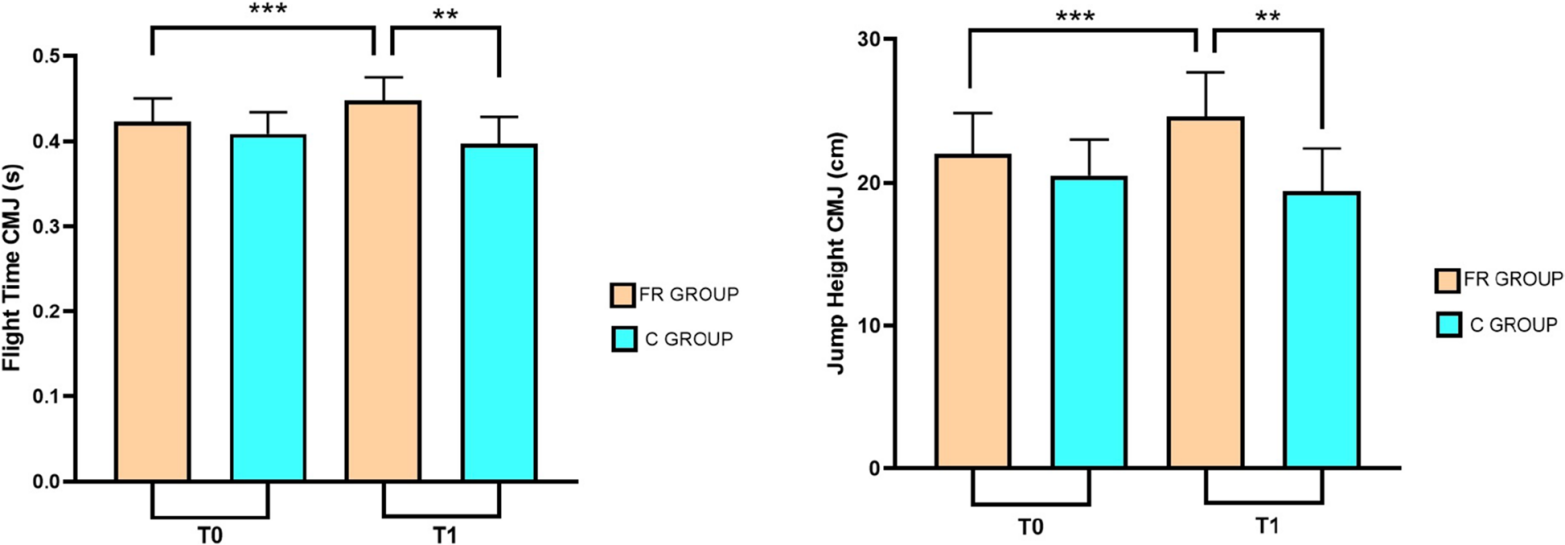
Differences in jumping ability before (T0) and after (T1) the interventions for both groups

Twenty-six elite young female rhythmic gymnasts completed the intervention and were included in the study for analysis. Participants were allocated into two groups: the FR (Foam Rolling) group, comprising 12 subjects, and the C (Control) group, comprising 14 subjects. A Post hoc analysis sample size power was calculated for a repeated-measures ANOVA using G*Power 3.1 software [34]. The analysis, with an effect size of 0.30 and a type I error rate of 0.05, achieved a power of 0.84. The anthropometric characteristics of the participants in both groups are shown in Table 1. While no significant baseline differences were observed between groups regarding age, height, or body mass, the FR group exhibited significant temporal changes from T0 to T1. Indeed, the repeated measures ANOVA showed significant between-subject interaction effects (F = 32.58; *p <* 0.001; ηp^2^ = 0.576), and group-by-time interaction effects (F = 9.73; *p <* 0.01; ηp^2^ = 0.288). A detailed post-hoc analysis showed, through Table 2, Figs. 3 and 4, which variables improved in the FR group compared to the C group. The FR group showed significant differences between T0 and T1 exclusively in CMJ performance: Flight Time (*p <* 0.001) and Jump Height (*p <* 0.001). Regarding the differences between the FR group and the C group in T1, an increase in the angular velocity of dorsiflexion of the right ankle (*p <* 0.05) and an increase in CMJ values (*p <* 0.01) were found.

**Table 1:** Anthropometric results and Student’s t-test results for respective variables

	**FR Group**	**C Group**	p*
Participants (n)	12	14	ns
Age (years)	13.2 ± 2.59	13.4 ± 2.17	ns
Height (cm)	152.3 ± 13.34	149.5 ± 13.33	ns
Weight (kg)	39.9 ± 11.22	40.6 ± 10.14	ns

Legend. *ns* not significant

^*^p < 0.05; **p < 0.01; ***p < 0.001

**Table 2:** Measurements before (T0) and after (T1) the conditions

		**FR Group (*n =* 12)**		**C Group (*n =* 14)**	
		**T0**	**T1**	**T0**	**T1**
Left-Dorsiflexion	ROM (°)	29.1 ± 7.86	28.9 ± 7.56	28.8 ± 6.32	25.7 ± 4.6
	Angular speed (°/s)	54.8 ± 29.24	62.9 ± 27.75	45 ± 15.91	47.9 ± 17.33
	Fluency Index	0.938 ± 0.068	0.898 ± 0.09	0.906 ± 0.093	0.916 ± 0.057
Right-Dorsiflexion	ROM (°)	27.8 ± 4.92	30.8 ± 5.3	26 ± 5.77	26 ± 4.7
	Angular speed (°/s)	50.1 ± 17.67	57.5 ± 25.33^*^	47.6 ± 23.29	37.4 ± 8.26
	Fluency Index	0.942 ± 0.074	0.876 ± 0.09	0.916 ± 0.06	0.879 ± 0.09
Flight Time CMJ (s)		0.423 ± 0.027	0.448 ± 0.027^***^	0.408 ± 0.025	0.397 ± 0.03
Jump Height CMJ (cm)		22.017 ± 2.84	24.63 ± 3.09^***^	20.5 ± 2.53	19.44 ± 2.95

Legend. *ns* not significant

^*^p < 0.05; **p < 0.01; ***p < 0.001

## Discussion

This study evaluated the effects of 3 weeks of FR during the Warm-Up phase on ankle dorsiflexion parameters and leaping ability in young rhythmic gymnasts. The protocol used showed positive effects on the ankle dorsiflexion parameters analyzed. However, only the angular velocity of the right ankle showed a statistically significant difference between the two groups, in contrast to the ROM and fluency index. Interestingly, the intervention appeared to exert a protective effect on the left ankle, mitigating the decline in performance observed in the control group. Conversely, it facilitated an improvement in the right ankle, suggesting a differentiated response between limbs. This phenomenon could be explained by the characteristics of rhythmic gymnastics. The asymmetrical load inherent during activity could result from the continuous repetition of unilateral tasks. Frequent jumps and landings, combined with the continuous use of a dominant leg to ensure stability during rotations, could promote lateralized adaptations in neuromuscular control during critical phases of physical development. Nevertheless, further studies are needed to evaluate motor control and movement asymmetries in rhythmic gymnastics. Several studies in the literature examined the acute effects of FR during warm-up, finding an increase in joint ROM, performance and other parameters such as muscle stiffness and neuromuscular control [35–37]. However, to the best of the authors' knowledge, few studies have examined the long-term effects of FR, and existing studies have not taken FR into account during the warm-up phase. Furthermore, they also differ in terms of the period of administration. In this regard, Hodgson et al. in 2018 evaluated the effects of FR for a 4-week intervention against a control group [38]. The authors used a FR protocol, with three sessions per week consisting of four sets of 30 s on the quadriceps and hamstrings of the dominant limb [38]. This treatment did not lead to any significant changes in ROM and jumping ability, indicating that FR leads to acute benefits rather than medium- to long-term benefits [38]. In contrast, Smith et al. evaluated statistically significant effects of a 6-week FR protocol with a frequency of twice a week on ankle ROM, finding results similar to static stretching [39]. At the same time, Kasahara et al. obtained significant results on ROM in ankle dorsiflexion after a 6-week FR protocol, finding no difference with vibration foam rolling [40]. Similarly, using a 5-week intervention, Kiyono et al. found significant effects on ROM [41], indicating the effectiveness of FR protocols lasting one week less than in previous studies. Indeed, recent studies have investigated the chronic effects of FR on joint ROM, suggesting a protocol lasting more than 4 weeks to create adaptation in the muscle–tendon structures [42, 43]. Our results partly confirm those reported in the literature. A 3-week intervention was not sufficient to create a significant adaptation in ankle ROM, although the increase in angular velocity could indicate the beginning of the process. It is plausible that a more prolonged intervention period would have yielded significant improvements in ankle joint ROM [42]. Consequently, future longitudinal studies are warranted to establish the minimum effective duration required to elicit stable structural and functional adaptations in this specific population. However, to the best of our knowledge, this is the first study to evaluate an FR protocol inside a warm-up and to establish its medium- to long-term effects. About CMJ values, it is interesting to note that the control group recorded a deterioration in vertical jump performance, in contrast to the FR group. This phenomenon could possibly be influenced by seasonal training load linked to the competitive period, suggesting that the foam roller may also act as a protective factor against overload stress. Nevertheless, these considerations remain purely theoretical due to the lack of training load monitoring. Although many studies in the literature have found a relationship between increased joint ROM parameters and jump height, this study, conducted on young rhythmic gymnasts, does not appear to support this finding. However, it should be noted that this study examined a specific population of athletes who undergo long training sessions characterized by exercises that require high muscle flexibility and repeated jumps. Therefore, these results should be interpreted in the context of rhythmic gymnastics performance. Furthermore, the increase in vertical jump could be a neuromuscular consequence of warm-up in general and FR in particular. Indeed, some studies have shown the effects of myofascial release on muscle fiber recruitment, reducing adhesions and thus improving power expression [44, 45]. Therefore, the increase in the angular velocity of right dorsiflexion movement may have been a contributing factor towards the improvement in the jumping ability of the young gymnasts. Nevertheless, further studies are needed to investigate motor control following warm-up and FR. This study is not lacking in limitations. Firstly, a randomized trial was not conducted. However, this approach was intentionally adopted to maintain the integrity of training groups in the elite youth environment and avoid interfering with established training protocols. Although this ensured high athlete adherence, the lack of randomized assignment may have introduced selection bias, potentially limiting the ability to draw definitive causal conclusions. Secondly, the small sample size may limit statistical power and stability of effect-size estimates. While the post hoc analysis indicated a power of 0.84, the absence of an a priori sample size estimation remains a methodological limitation that warrants caution in the interpretation of these findings. Thirdly, only female rhythmic gymnasts were recruited, who are nonetheless representative of an Olympic discipline practiced by young women and therefore contextualizable to this type of population. Further research is warranted to assess the efficacy of FR as a warm-up modality across different populations. Future studies should include male cohorts, broader age ranges, and athletes from various sporting disciplines to enhance the generalizability of these findings.

### Practical implication

Several practical applications can be drawn from this study. The warm-up of athletes is a fundamental activity for maximizing their sports performance. Confirming the results of this study, foam rolling is a potentially effective non-invasive tool for warming up athletes that could be incorporated into their routine. Prolonged use of foam rolling before training could enhance jumping performance. This study may also be useful for rhythmic gymnastics coaches and trainers to maximize athletic jumping performance and may support performance maintenance.

## Conclusions

In conclusion, our results affirmed that adding a 3-week FR protocol during warm-up increased jumping ability in young rhythmic gymnasts. Furthermore, this protocol did not produce significant effects on ankle dorsiflexion parameters: ROM, angular velocity, and fluency index, except for right ankle angular velocity. Therefore, based on the results of this study, three weeks of FR during the warm-up phase would appear to be sufficient to influence CMJ height, but not ankle dorsiflexion ROM in young rhythmic gymnasts. Future studies should evaluate longer intervention protocols to support the existing literature [42, 43].

## Data Availability

The data presented in this study are available on request from the corresponding author.
